# Oxidation by trace Cu^2+^ ions underlies the ability of ascorbate to induce vascular dysfunction in the rat perfused mesentery

**DOI:** 10.1016/j.ejphar.2009.04.033

**Published:** 2009-07-01

**Authors:** Silvia Nelli, John Craig, William Martin

**Affiliations:** Integrative & Systems Biology, Faculty of Biomedical & Life Sciences, West Medical Building, University of Glasgow, Glasgow, G12 8QQ, Scotland, UK

**Keywords:** Ascorbate, Copper, EDHF, Endothelium-derived hyperpolarizing factor, Hydrogen peroxide, Oxidant stress

## Abstract

Ascorbate has both antioxidant and pro-oxidant activities. We have previously shown that plasma levels of ascorbate induce constriction and blockade of dilatation mediated by endothelium-derived hyperpolarizing factor (EDHF). In this study we sought to determine if these detrimental actions were mediated by a pro-oxidant action of ascorbate. Since trace levels of transition metal ions including, Cu^2+^ and Fe^3+^, promote oxidation of ascorbate, we examined the effects of the chelating agents, cuprizone and deferoxamine, and of CuSO_4_ and FeCl_3_ on ascorbate-induced constriction and blockade of EDHF in the perfused rat mesentery. Cuprizone abolished and Cu^2+^ but not Fe^3+^ ions enhanced both ascorbate (50 μM)-induced constriction and blockade of EDHF. The blockade of EDHF produced by ascorbate in the presence of CuSO_4_ (0.5 μM) was abolished by the hydrogen peroxide scavenger, catalase, but unaffected by the scavengers of hydroxyl radical or superoxide anion, mannitol and superoxide dismutase (SOD), respectively. Consistent with these observations, the oxidation of ascorbate by CuSO_4_ led to the rapid production of hydrogen peroxide. Catalase, mannitol and SOD had no effect on ascorbate-induced constriction. Thus, in the rat perfused mesentery, both the constrictor and EDHF-blocking actions of ascorbate arise from its oxidation by trace Cu^2+^ ions. The blockade of EDHF results from the consequent generation of hydrogen peroxide, but the factor producing constriction remains unidentified. These detrimental actions of ascorbate may help explain the disappointing outcome of clinical trials investigating dietary supplementation with the vitamin on cardiovascular health.

## 1 Introduction

Ascorbate (or vitamin C) plays a vital role in a wide variety of biological functions, including the synthesis of collagen, carnitine and amine transmitters, and is the most important water-soluble antioxidant in humans (Frei, 1994). In the cardiovascular system, the effects of ascorbate on nitric oxide-mediated vasodilatation are well studied (Dudgeon et al., 1998; Fontana et al., 1999; Carr et al., 2000; Fontana et al., 1999). These reports demonstrate that ascorbate is able to restore nitric oxide-dependent vasodilatation following its impairment by oxidant stress. Whether this protective action results from the ability of ascorbate to scavenge superoxide anion and so prevent it from destroying nitric oxide (Gryglewski et al., 1986; Rubanyi and Vanhoutte, 1986) or from some other mechanism (Jackson et al., 1998) remains unresolved. Nevertheless, there are many reports where acute treatment with ascorbate improves the impaired nitric oxide-mediated dilatation in patients with a variety of cardiovascular pathologies, including essential hypertension (Taddei et al., 1998; Natali et al., 2000), atherosclerosis (Levine et al., 1996; Ting et al., 1997) and heart failure (Ellis et al., 2001; Hornig et al., 1998). Despite this, the outcome of clinical trials studying the effects of dietary supplementation with ascorbate on a range of cardiovascular endpoints has been disappointing (Collins et al., 2002; Duarte and Lunec, 2005). It is possible, therefore, that any benefit arising from the ability of ascorbate to enhance the activity of nitric oxide is offset by a detrimental pro-oxidant action (Duarte and Lunec, 2005).

We have previously shown in the rat perfused mesentery and bovine perfused ciliary artery that plasma levels of ascorbate (10–150 μM) produce two potentially detrimental actions: a constrictor action, rapid in the former but slowly developing in the latter, together with a time-dependent blockade (2–3 h max) of dilatation mediated by endothelium-derived hyperpolarizing factor (EDHF) in both tissues (Stirrat et al., 2006; Stirrat et al., 2008; McNeish et al., 2002). Furthermore, the inhibition of EDHF induced by ascorbate appears highly selective, since the vasodilator actions of endothelium-derived nitric oxide, the nitrovasodilator, glyceryl trinitrate, and the K_ATP_ channel opener, levcromakalim, remain entirely unaffected. We and others have shown, however, that very high concentrations of ascorbate (3–10 mM) impair acetylcholine-induced, nitric oxide-mediated dilatation in the rabbit aorta (de Saram et al., 2002; McNeish et al., 2003).

The aim of this study was to determine if the constrictor and EDHF-blocking actions seen in the rat perfused mesentery by plasma levels of ascorbate could be explained by a pro-oxidant action. Indeed, the findings show that oxidation of ascorbate by trace Cu^2+^ ions is responsible for both detrimental vascular actions.

## 2 Materials and methods

### 2.1 Preparation of the rat perfused mesentery for pressure recording

All animal procedures were conducted according to the ethical guidelines of the University of Glasgow under Schedule 1 of the Animals (Scientific Procedures) Act 1986. Male Wistar rats (150–180 g) were killed by concussion followed by exsanguination. The superior mesenteric artery was cannulated and the mesenteric arterial vasculature dissected from the intestines and suspended in a heated organ bath as previously described (McNeish et al., 2002; Stirrat et al., 2008). Mesenteries were then perfused at 37 °C using a peristaltic pump (Minipuls 3, Gilson) at a flow rate of 15 ml/min with Krebs solution containing (mM): NaCl, 118; KCl, 4.7; CaCl_2_, 2.5; KH_2_PO_4_, 1.2; MgSO_4_, 1.2; NaHCO_3_, 25; glucose, 11.5; and gassed with 95% O_2_/5% CO_2_. Tissues were allowed to equilibrate for at least 30 min before the beginning of each experiment. Perfusion pressure was measured using Gould Statham P32 ID transducers via a side arm located immediately proximal to the inflow cannula and displayed on a PowerLab data acquisition system (AD Instruments, Hastings, U.K.).

### 2.2 Experimental protocols with the rat perfused mesentery

In order to observe EDHF-mediated dilator responses to acetylcholine, the perfusion pressure was raised to ~ 120 mm Hg using the thromboxane-mimetic, U46619 (10–100 nM). The effects of nitric oxide and cyclooxygenase products were routinely blocked using N^G^-nitro-l-arginine methyl ester (L-NAME, 100 μM) and indomethacin (3 μM), respectively. Vasodilator responses (~ 50% max) to acetylcholine (10 nmol) were elicited at 15 min intervals during a 3 h study period by injecting into the perfusion fluid 10 μl volumes of stock solution using a Hamilton micro-syringe, and expressed as a percentage of the U46619-induced perfusion pressure.

As will be seen in the Results, the ability of ascorbate (50 μM) to induce a rapid constriction and a slowly developing (max at 3 h) blockade of acetylcholine-induced, EDHF-mediated dilatation (Stirrat et al., 2008) was abolished by the Cu^2+^ ion chelator, cuprizone (30 μM), and enhanced by CuSO_4_ (0.5 μM). As a consequence, except when otherwise stated, all further experiments with ascorbate (50 μM) were conducted on tissues perfused with Krebs containing CuSO_4_ (0.5 μM). A number of agents were investigated for their ability to inhibit the constrictor and EDHF-blocking actions of ascorbate in the presence of CuSO_4_. These were: the superoxide scavengers, superoxide dismutase (SOD, 100 u/ml), tiron (300 μM) and Tempol (1 mM); the NADPH oxidase inhibitor, diphenyleneiodonium (3 μM); the xanthine oxidase inhibitor, allopurinol (100 μM); the mitochondrial inhibitors, rotenone (1 μM) and myxothiazole (0.3 μM); the hydrogen peroxide scavenger, catalase (1200 u/ml); and the hydroxyl radical scavenger, mannitol (10 mM).

Time-matched control experiments were conducted in the absence of ascorbate and CuSO_4_ to determine the reproducibility of the dilator action of acetylcholine. In addition, the selectivity of the blockade of acetylcholine-induced, EDHF-mediated vasodilatation by ascorbate in the presence of CuSO_4_ was assessed by examining the effects on dilatation to the endothelium-independent agent, levcromakalim (5 nmol).

Experiments were conducted to determine if FeCl_3_ (0.5 and 100 μM) could mimic the ability of CuSO_4_ to facilitate ascorbate (50 μM)-induced constriction and blockade of acetylcholine-induced, EDHF-mediated dilatation. Before these experiments were conducted, Cu^2+^-free conditions were produced by purging the perfusion system with Krebs containing cuprizone (30 μM). Cuprizone was not, however, present during these experiments to guard against any possibility of chelation of Fe^3+^ ions.

Other experiments were conducted to determine if hydrogen peroxide (30 and 50 μM) could mimic the ability of ascorbate to induce constriction and blockade of acetylcholine-induced, EDHF-mediated dilatation. These experiments were conducted in the presence of cuprizone (30 μM) to ensure any effects seen were due to hydrogen peroxide per se and not to other reactive oxygen species formed following its interaction with trace Cu^2+^ ions.

### 2.3 Assay of hydrogen peroxide formation

The ability of Cu^2+^ and Fe^3+^ ions to catalyse the formation of hydrogen peroxide from ascorbate was assessed using the FOX2 (ferrous oxidation in xylenol orange, version 2) method (Nourooz-Zadeh et al., 1994). The FOX2 reagent was prepared by adding one volume of solution 1 (1 mM xylenol orange and 2.5 mM ammonium ferrous sulphate in 250 mM sulphuric acid) to 9 volumes of solution 2 (4.4 mM butylated hydroxytoluene in HPLC grade methanol). Experiments were carried out at 37 °C in organ baths filled with the same Krebs solution used for the biological experiments and gassed with 95% O_2_/5% CO_2_·CuSO_4_ (0.5 μM) or FeCl_3_ (100 μM) was added to the Krebs solution and 100 μl samples (time zero) taken for assay. Ascorbate (50 μM) was then added to the Krebs solution and 100 μl samples taken for assay at 2, 30, 60, 120 and 180 min. Once taken, samples were immediately added to 1 ml of FOX2 reagent and incubated at room temperature for 30 min. Absorbance at 560 nm was read using a Pye Unicam SP6-550 spectrophotometer, with hydrogen peroxide levels assessed using standards of known concentration (0–100 μM). Fresh standards and blanks were prepared for each time point because their absorbance did not remain stable during the 3 h duration of experiments.

### 2.4 Drugs and chemicals

Acetylcholine chloride, ammonium ferrous sulphate, allopurinol, ascorbic acid, butylated hydroxytoluene, cuprizone, deferoxamine, diphenyleneiodonium, indomethacin, myxothiazole, l-NAME (N^G^-nitro-l-arginine methyl ester), mannitol, rotenone, Tempol, tiron and U46619 (9,11-dideoxy-11α,9α-epoxy-methanoprostaglandin F_2α_) were all obtained from Sigma. Catalase (bovine liver) was obtained from Calbiochem. Hydrogen peroxide was obtained from BDH. Levcromakalim was a gift from GlaxoSmithKline (Harlow, UK). Xylenol orange disodium salt was obtained from Fluka. All drugs were dissolved and diluted in 0.9% saline except indomethacin (1 mM stock), which was dissolved in Na_2_CO_3_ (0.4 mg/ml), rotenone (10 mM stock in ethanol), levcromakalim (0.1 M stock in 70% ethanol), cuprizone and U46619 (10 mM and 1 mM stocks, respectively, in 50% ethanol), allopurinol, diphenyleneiodonium and myxothiazole (0.1 M, 30 mM and 3 mM stocks, respectively, in DMSO).

### 2.5 Statistical analysis

Results are expressed as the mean ± S.E.M. of *n* separate observations, each from a separate tissue. Graphs were drawn and statistical comparisons made using one-way analysis of variance and Bonferroni's post-test with the aid of a computer program, Prism (GraphPad, San Diego, USA). A probability (*P*) less than or equal to 0.05 was considered significant.

## 3 Results

### 3.1 Effects of ascorbate on the rat perfused mesentery in the presence of Cu^2+^ or Fe^3+^ ions

**Fig. 1:**
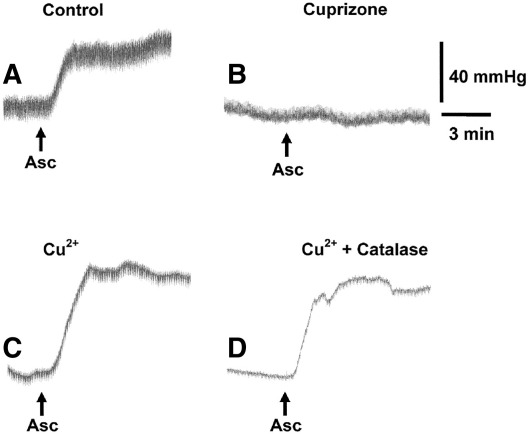
Experimental traces showing: (A) the ability of ascorbate (50 μM) to induce constriction in the rat perfused mesentery; (B) the abolition of this constriction in the presence of the Cu^2+^ chelator, cuprizone (30 μM); (C) the enhancement of ascorbate-induced constriction in the presence of CuSO_4_ (0.5 μM); and (D) the inability of catalase (1200 u/ml) to inhibit ascorbate-induced constriction.

**Fig. 2:**
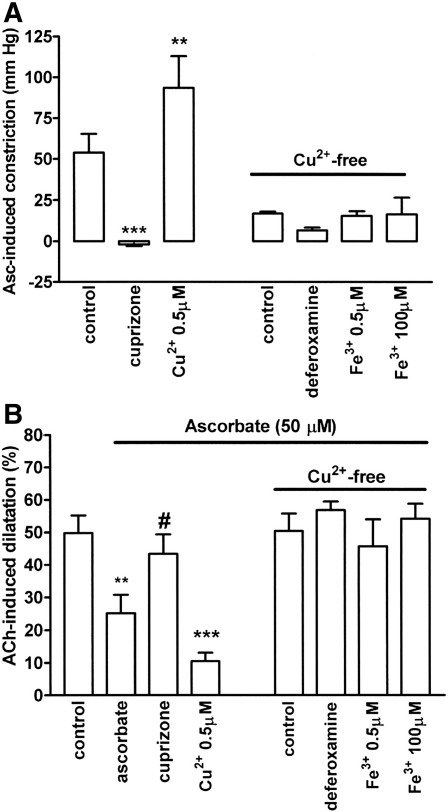
The Cu^2+^ chelator, cuprizone (30 μM), abolishes and CuSO_4_ (0.5 μM) enhances the ability of ascorbate (50 μM) to (A) induce constriction and (B) inhibit acetylcholine (10 nmol)-induced, EDHF-mediated dilatation in the rat perfused mesentery. Following prior purging of the perfusion system with cuprizone (Cu^2+^-free), ascorbate induced only modest constriction and failed to inhibit acetylcholine-induced dilatation in the presence of FeCl_3_ (0.5 and 100 μM) or the Fe^3+^ chelator, deferoxamine (100 μM). Data are mean ± S.E.M. of 6–9 observations. ⁎⁎*P* < 0.01 and ⁎⁎⁎*P* < 0.001 indicate significant differences from respective controls. ^#^*P* < 0.05 indicates a significant restoration of acetylcholine-induced dilatation.

In the rat mesenteric vascular bed, perfused at 15 ml/min in the presence of L-NAME (100 μM) and indomethacin (3 μM), perfusion pressure was raised to ~ 120 mm Hg using U46619 (10–100 nM). Under these conditions, acetylcholine (10 nmol) induced powerful EDHF-mediated dilatation (49.8 ± 5.4%, *n* = 8; Fig. 2). As previously reported (Stirrat et al., 2008), treatment with ascorbate (50 μM) induced a rapid rise in perfusion pressure (53.8 ± 11.8 mm Hg, *n* = 9; Figs. 1 and 2) and a slowly developing blockade of acetylcholine-induced, EDHF-mediated dilatation (reduced to 25.2 ± 5.6% at 3 h, *n* = 7, Fig. 2). The Cu^2+^ chelator, cuprizone (30 μM), abolished the ability of ascorbate to induce constriction and block acetylcholine-induced dilatation (Figs. 1 and 2). Moreover, treatment with CuSO_4_ (0.5 μM) enhanced the ability of ascorbate to induce constriction and block acetylcholine-induced dilatation. In contrast, following prior purging of the perfusion system with cuprizone (30 μM) to create Cu^2+^-free conditions, FeCl_3_ (0.5 and 100 μM) lacked the ability of Cu^2+^ to support ascorbate-induced constriction or blockade of acetylcholine-induced dilatation. In addition, the Fe^3+^ chelator, deferoxamine (100 μM), had no statistically significant influence on the poor ability of ascorbate to induce constriction or blockade of acetylcholine-induced dilatation under these conditions.

### 3.2 Selectivity of the blockade of EDHF-mediated dilatation by ascorbate in the presence of Cu^2+^ ions

**Fig. 3:**
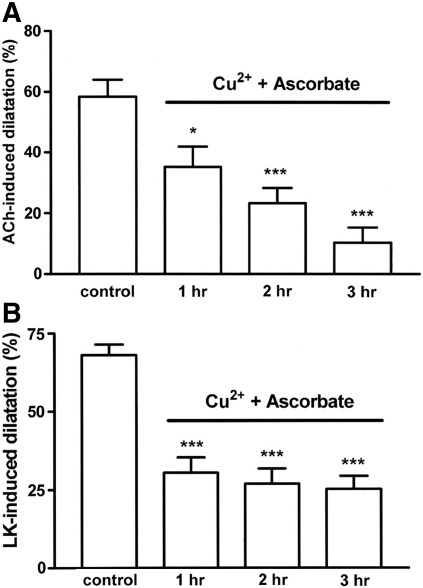
Timecourse showing the ability of ascorbate (50 μM) in the presence of CuSO_4_ (0.5 μM) to inhibit (A) acetylcholine (10 nmol)-induced, EDHF-mediated dilatation and (B) levcromakalim (5 nmol)-induced dilatation in the rat perfused mesentery during a 3 h incubation. Data are mean ± S.E.M. of 6–10 observations. ⁎*P* < 0.05 and ⁎⁎⁎*P* < 0.001 indicate significant differences from respective controls.

Our previous findings with ascorbate on the rat mesentery showed that it produced selective blockade of acetylcholine-induced, EDHF-mediated dilatation, with no effects on the endothelium-independent dilatation induced by levcromakalim (Stirrat et al., 2008). Fig. 3 shows, however, that in the additional presence of CuSO_4_ (0.5 μM), which enhances the time-dependent blockade of acetylcholine (10 nmol)-induced dilatation, vasodilator responses to levcromakalim (5 nmol) were also inhibited.

### 3.3 Involvement of reactive oxygen species in the actions of ascorbate

**Fig. 4:**
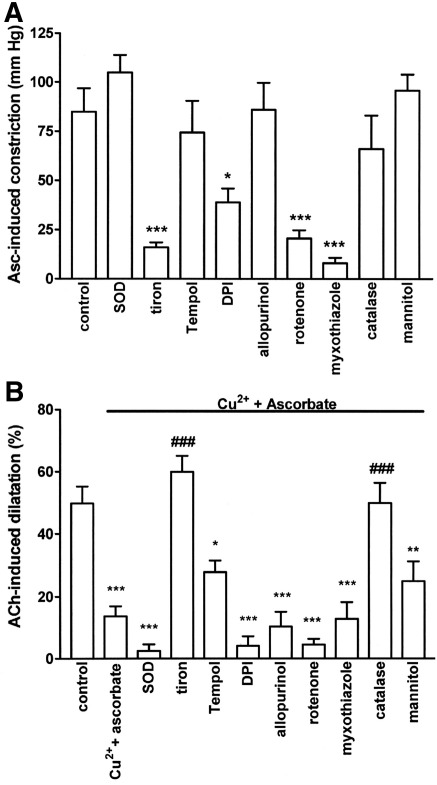
The ability of ascorbate (50 μM) in the presence of CuSO_4_ (0.5 μM) to (A) induce constriction and (B) inhibit acetylcholine (10 nmol)-induced, EDHF-mediated dilatation in the rat perfused mesentery and the effects on these responses produced by prior treatment with superoxide dismutase (SOD, 100 u/ml), tiron (300 μM), Tempol (1 mM), diphenyleneiodonium (DPI, 3 μM), allopurinol (100 μM), rotenone (1 μM), myxothiazole (0.3 μM), catalase (1200 u/ml) and mannitol (10 mM). Data are mean ± S.E.M. of 5–9 observations. ⁎*P* < 0.05, ⁎⁎*P* < 0.01 and ⁎⁎⁎*P* < 0.001 indicate significant differences from respective controls. ^###^*P* < 0.001 indicates a significant restoration of acetylcholine-induced dilatation.

The ability of ascorbate (50 μM) in the presence of CuSO_4_ (0.5 μM) to induce both constriction and blockade of acetylcholine (10 nmol)-induced, EDHF-mediated dilatation was unaffected by the superoxide scavengers, SOD (100 u/ml) and Tempol (1 mM), but was abolished by tiron (300 μM; Fig. 4). The NADPH oxidase inhibitor, diphenyleneiodonium (3 μM), and the mitochondrial inhibitors, rotenone (1 μM) and myxothiazole (0.3 μM), each induced substantial falls in U46619-induced perfusion pressure (of 81.2 ± 11.5, 32.7 ± 12.0 and 24.8 ± 9.0 mm Hg, respectively, *n* = 5–6). They each also depressed the ability of ascorbate to induce constriction, but had no effect on its blockade of acetylcholine-induced dilatation (Fig. 4). Neither the xanthine oxidase inhibitor, allopurinol (100 μM), nor the hydroxyl radical scavenger, mannitol (10 mM), had any effect on ascorbate-induced constriction or the blockade of acetylcholine-induced dilatation. The hydrogen peroxide scavenger, catalase (1200 u/ml), had no effect on ascorbate-induced constriction, but abolished the blockade of acetylcholine-induced dilatation (Figs. 1 and 4).

### 3.4 Generation of hydrogen peroxide following the oxidation of ascorbate

**Fig. 5:**
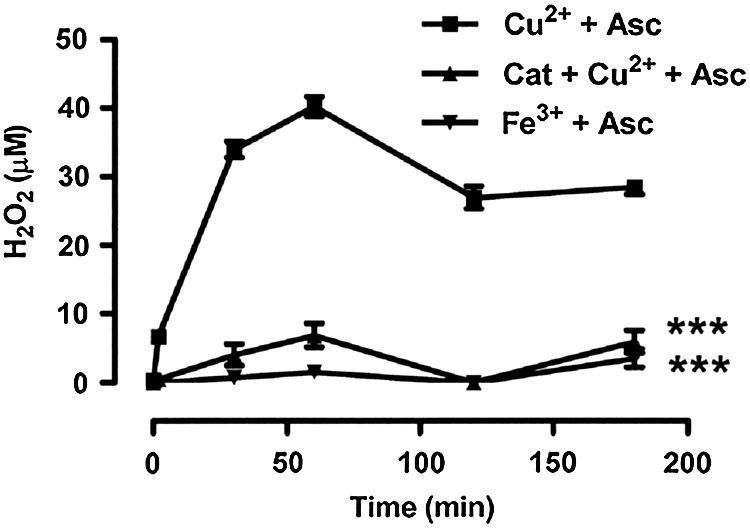
Timecourse showing the generation of hydrogen peroxide during the oxidation of ascorbate (50 μM) by CuSO_4_ (0.5 μM) in Krebs solution at 37 °C. Catalase (1200 u/ml) prevented this accumulation of hydrogen peroxide. FeCl_3_ (100 μM) failed to generate appreciable levels of hydrogen peroxide from ascorbate. Data are mean ± S.E.M. of 5–6 observations. ⁎⁎⁎*P* < 0.001 indicates a significant difference from the hydrogen peroxide levels generated by CuSO_4_ and ascorbate.

The ability of catalase to inhibit the blockade of acetylcholine-induced dilatation by ascorbate in the presence of CuSO_4_ prompted us to examine the generation of hydrogen peroxide. In the absence of tissues, the addition of ascorbate (50 μM) to Krebs solution containing CuSO_4_ (0.5 μM) at 37 °C led to the rapid generation of hydrogen peroxide (Fig. 5): 6.6 ± 1.8 μM (*n* = 6) was detected at 2 min and a maximum of 40.2 ± 3.6 μM was seen at 1 h, with only a slight decline during the remainder of the 3 h incubation. The accumulation of hydrogen peroxide under these conditions was almost abolished by catalase (1200 u/ml). In contrast to CuSO_4_, FeCl_3_ (100 μM) failed to generate appreciable levels of hydrogen peroxide from ascorbate.

### 3.5 Effects of hydrogen peroxide on the rat mesentery

**Fig. 6:**
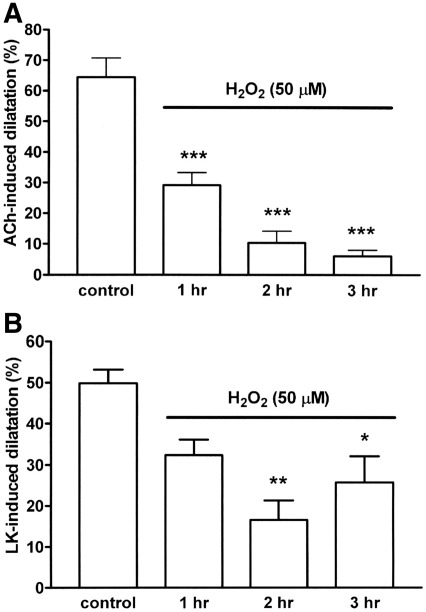
Timecourse showing the ability of hydrogen peroxide (50 μM) to inhibit (A) acetylcholine (10 nmol)-induced, EDHF-mediated dilatation and (B) levcromakalim (5 nmol)-induced dilatation in the rat perfused mesentery during a 3 h incubation. Data are mean ± S.E.M. of 5–7 observations. ⁎*P* < 0.05, ⁎⁎*P* < 0.01 and ⁎⁎⁎*P* < 0.001 indicate significant differences from respective controls.

The generation of hydrogen peroxide during the oxidation of ascorbate by Cu^2+^, coupled with the ability of catalase to inhibit the associated blockade of acetylcholine-induced dilatation but not the constriction, led us to examine which of these vascular actions could be mimicked by exogenous hydrogen peroxide. We found that hydrogen peroxide failed to produce constriction of the rat mesentery at any concentration tested (1–100 μM). It did, however, induce dilatations of 13.9 ± 2.8%, 23.0 ± 6.5% and 76.7 ± 21.2% (*n* = 5), at concentrations of 10, 30 and 100 μM respectively. Hydrogen peroxide (30 μM) produced a small but significant blockade of acetylcholine (10 nmol)-induced dilatation at 3 h (control 49.8 ± 5.5%; treated 28.2 ± 4.0%, *n* = 6, *P* < 0.05), but had no significant effect on dilatation to levcromakalim (5 nmol; control 48.7 ± 7.1%; treated 38.7 ± 4.1%, *n* = 9). Raising the hydrogen peroxide concentration to 50 μM led to a time-dependent blockade of acetylcholine-induced dilatation and blocked levcromakalim-induced dilatation at 2 and 3 h (Fig. 6).

## 4 Discussion

The most striking new finding in this study is that the Cu^2+^ chelator, cuprizone (Peterson and Bollier, 1955; Gordge et al., 1995), abolishes the ability of plasma levels of ascorbate (50 μM) to produce a rapid constriction and slowly developing (over 2–3 h) blockade of acetylcholine-induced, EDHF-mediated dilatation in the rat perfused mesentery (McNeish et al., 2002; Stirrat et al., 2008). Moreover, a low concentration of CuSO_4_ (0.5 μM) augmented the ability of ascorbate to induce constriction and block EDHF. These findings suggest that trace levels of Cu^2+^ ions are required to uncover these vascular actions of ascorbate.

Transition metal ions commonly encountered in biology, namely, Cu^2+^ and Fe^3+^, can catalyse the oxidation of ascorbate (Rowley and Halliwell, 1983; Aruoma et al., 1991). These metal ions are present in the vascular wall and may play a role in cardiovascular disease (Evans et al., 1995; Rajendran et al., 2007), but trace levels also contaminate even the most carefully prepared physiological solutions and buffers, commonly reaching ~ 0.1 μM for Cu^2+^ and 1–10 μM for Fe^3+^ (Buettner and Jurkiewicz, 1996; Van der Zee and Van den Broek, 1998). We found, however, that of the two metal ions, only Cu^2+^ promoted the ability of ascorbate to induce constriction and block EDHF; Fe^3+^ was ineffective, even at concentrations as high as 100 μM. Furthermore, our experiments conducted after prior purging of the perfusion system with cuprizone resulted in only slightly less blockade of ascorbate's actions than those in which cuprizone was actually present (Fig. 2). We therefore suspect that trace levels of Cu^2+^ in the Krebs solution are mainly responsible for the vascular actions of ascorbate, with perhaps a small contribution from tissue-derived sources.

There is debate about whether or not oxidation of ascorbate generates superoxide anion (Halliwell and Foyer, 1976; Scarpa et al., 1983), but there is general agreement that oxidation by Cu^2+^ or Fe^3+^ will, providing a source of hydrogen peroxide is available, result in the generation of hydroxyl radical (HO^•^), according to the following scheme (Scarpa et al., 1983; Rowley and Halliwell, 1983; Aruoma et al., 1991):

(1) ascorbate + Cu^2+^ (or Fe^3+^) → ascorbyl radical + Cu^+^ (or Fe^2+^) + H^+^

(2) H_2_O_2_ + Cu^+^ (or Fe^2+^) + H^+^ → HO^•^ + H_2_O + Cu^2+^ (or Fe^3+^).

We therefore examined the effects of scavengers of different reactive oxygen species on the ability of ascorbate (in the presence of 0.5 μM CuSO_4_) to induce constriction and blockade of EDHF. Our most significant finding was that catalase abolishes the ability of ascorbate to block EDHF-induced dilatation, strongly suggesting the involvement of hydrogen peroxide. In contrast, mannitol (Aruoma et al., 1991) was inactive, demonstrating that the catalase-sensitive blockade of EDHF by ascorbate did not require the conversion of hydrogen peroxide to hydroxyl radical. As hydrogen peroxide is commonly derived from superoxide anion, we investigated possible sources of these reactive oxygen species. Since the ability of ascorbate both to promote constriction and blockade of EDHF is flow-dependent (Nelli et al., 2004; Stirrat et al., 2006; Stirrat et al., 2008), we initially investigated sources of superoxide/hydrogen peroxide that are stimulated by flow, such as endothelial NADPH oxidase (Hwang et al., 2003; Sorescu et al., 2004) and mitochondria (Liu et al., 2003). These potential sources were excluded, however, because the NADPH oxidase inhibitor, diphenyleneiodonium (Lassegue and Clempus, 2003), and the mitochondrial inhibitors, rotenone or myxothiazole (Weir et al., 1991; Griffith et al., 1987), failed to protect EDHF-induced dilatation from blockade by ascorbate. Xanthine oxidase too was ruled out as the source of superoxide/hydrogen because allopurinol (Sobey et al., 1993) failed to protect EDHF-induced dilatation. Nitric oxide synthase and cyclooxygenase were also excluded as sources since our experiments were routinely conducted in the presence of L-NAME and indomethacin. The superoxide scavenger, tiron, abolished the ability of ascorbate to block EDHF, but this almost certainly results from its ability to chelate Cu^2+^ ions (Majumder and Savariar, 1959), rather than its superoxide scavenging properties (Ledenev et al., 1986), because two other scavengers, SOD and Tempol (Wang et al., 1995), were ineffective. Thus, these experiments highlighted the involvement of hydrogen peroxide in the blockade of EDHF following Cu^2+^-induced oxidation of ascorbate, but failed to identify the source of this agent.

Several reactive oxygen species, including superoxide, hydrogen peroxide and hydroxyl radical, induce constriction in a variety of tissues (Shi et al., 2007; Gao and Lee, 2005; Shen et al., 2000). Nevertheless, our experiments with SOD, catalase and mannitol ruled out involvement of all three species in the constriction induced by ascorbate. The superoxide scavenger, tiron, powerfully inhibited constriction, but again, this probably results from its ability to chelate Cu^2+^ ions, rather than its superoxide scavenging properties, because SOD and Tempol, were ineffective. The NADPH oxidase inhibitor, diphenyleneiodonium, and the mitochondrial inhibitors, rotenone or myxothiazole, also reduced the magnitude of ascorbate-induced constriction, but these are likely to be non-specific actions because all three agents powerfully depressed U46619-induced tone too. Thus, although experiments with cuprizone clearly demonstrated that constriction of the rat mesentery induced by ascorbate requires its oxidation by trace Cu^2+^ ions, further studies are required to identify the constrictor agent responsible.

Our inability to identify a cellular source of the hydrogen peroxide involved in the ascorbate-induced blockade of EDHF prompted us to search elsewhere. Experiments with cells in culture, including vascular endothelial cells, show that the plasma levels of ascorbate (50 μM) used in our experiments produce no adverse effects, but much higher concentrations (mM) are cytotoxic (Clements et al., 2001; Varadharaj et al., 2005; Arakawa et al., 1994; Satoh and Sakagami, 1997). In common with our findings on the ascorbate-induced blockade of EDHF, this cytotoxicity was abolished by catalase and facilitated by Cu^2+^ but not Fe^3+^ ions. Both metal ions did, however, promote oxidation of ascorbate, as assessed by formation of ascorbyl radical (Satoh and Sakagami, 1997). Cytotoxicity was also associated with the accumulation of hydrogen peroxide in the culture medium, and indeed Cu^2+^ but not Fe^3+^ ions have been shown to generate ~ 500 μM hydrogen peroxide from 2 mM ascorbate in samples of water incubated at room temperature for several hours (Jansson et al., 2005).

We found, in a tissue bath containing Krebs solution at 37 °C that Cu^2+^ but not Fe^3+^ ions lead to a rapid, sustained and almost stoichiometric formation of hydrogen peroxide (~ 30–40 μM) from ascorbate (50 μM). We therefore sought to determine which of the vascular effects of ascorbate could be produced by these concentrations of exogenous hydrogen peroxide. Although hydrogen peroxide has been reported to produce constriction of the rat mesenteric artery at low concentrations (10–100 μM), with dilatation predominating at higher concentrations (Gao et al., 2003), we found that it failed to constrict the rat perfused mesentery at any concentration tested (1–100 μM) and produced only dilatation, small at 10–30 μM but substantial at 100 μM. Hydrogen peroxide at 30 μM, produced small but significant inhibition of acetylcholine-induced, EDHF-mediated dilatation, but raising the concentration to 50 μM, mimicked very well the time-dependent blockade induced by ascorbate during a 3 h incubation. These finding are consistent with our conclusion above from the use of catalase that the generation of hydrogen peroxide largely explains the ability of ascorbate to block EDHF, but plays no role in the constrictor action of ascorbate.

Although our previous experiments with ascorbate on the rat mesentery and bovine ciliary artery demonstrated that inhibition of EDHF was entirely selective, with no blockade of other vasodilator agents (McNeish et al., 2002; Nelli et al., 2004; Stirrat et al., 2008), augmenting its action in the present experiments with CuSO_4_ (0.5 μM), also led to blockade of the endothelium-independent dilator, levcromakalim. This outcome is perhaps unsurprising, given our new finding that hydrogen peroxide is responsible for the blockade induced by ascorbate and that high concentrations of this oxidant are known to produce vascular dysfunction (Mian and Martin, 1997; Ardanaz and Pagano, 2006). Our additional findings that 30 μM hydrogen peroxide selectively blocks dilatation to EDHF, whereas 50 μM blocks dilatation to both EDHF and levcromakalim, are consistent with this explanation. Clearly, however, the detrimental effects of hydrogen peroxide on EDHF highlighted here, contrast starkly with suggestions from others that the agent may either actually function as the EDHF or facilitate EDHF-induced dilatation in certain tissues (Miura et al., 2003; Shimokawa and Morikawa, 2005; Edwards et al., 2008).

In conclusion, our new findings show that oxidation by trace Cu^2+^ ions accounts for the ability of plasma levels of ascorbate to induce constriction and blockade of EDHF. The blockade of EDHF results from the ensuing generation of hydrogen peroxide, but the oxidation product causing constriction remains unidentified. These detrimental actions of ascorbate may help explain why clinical trials investigating the effects of dietary supplementation with ascorbate show no benefit to cardiovascular health.
